# Peculiarities of Glycerol Conversion to Chemicals Over Zeolite-Based Catalysts

**DOI:** 10.3389/fchem.2019.00233

**Published:** 2019-04-26

**Authors:** Oki Muraza

**Affiliations:** Center of Research Excellence in Nanotechnology and Chemical Engineering Department, King Fahd University of Petroleum and Minerals, Dhahran, Saudi Arabia

**Keywords:** Glycerol, biodiesel, aromatics, fuels, solid acid catalysts, hierarchical zeolites

## Abstract

Many countries have opted to produce biodiesel from vegetable oils for energy security and climate change concerns. Consequently, the availability of abundant glycerol, as a by-product in biodiesel production, is more obvious. Many institutions and companies have explored different routes to convert glycerol to highly-added chemical products and fuel additives. As the addition of the second reactant to glycerol may end up with worse exergy calculation, the conversion of glycerol over solid acid catalysts without the addition of the second reactant is preferred in this mini-review. Glycerol aromatization and glycerol dehydration over zeolite catalysts were focused with an emphasis on recent papers in the past 3 years. The role of acidity, hydrophilicity-hydrophobicity, zeolite frameworks are highlighted. The presence of water in the glycerol feed affected the stability of the catalysts. Low cost and naturally abundant zeolite and minerals are proposed. Numerous low-cost catalysts such as natural zeolites and natural clays are potentially used for this purpose.

## Introduction

Sustainability is one of the most important issues this century. Sustainable production of fuels and chemicals are among the most studied topics in heterogeneous catalysis. Diesel is an important fuel for transportation and industry. According to ExxonMobil Outlook for Energy in 2017 (Exxonmobil, 2017), the demand for diesel will grow to 30% due to the surge in diesel demand for trucks and ships.

**Figure 1:**
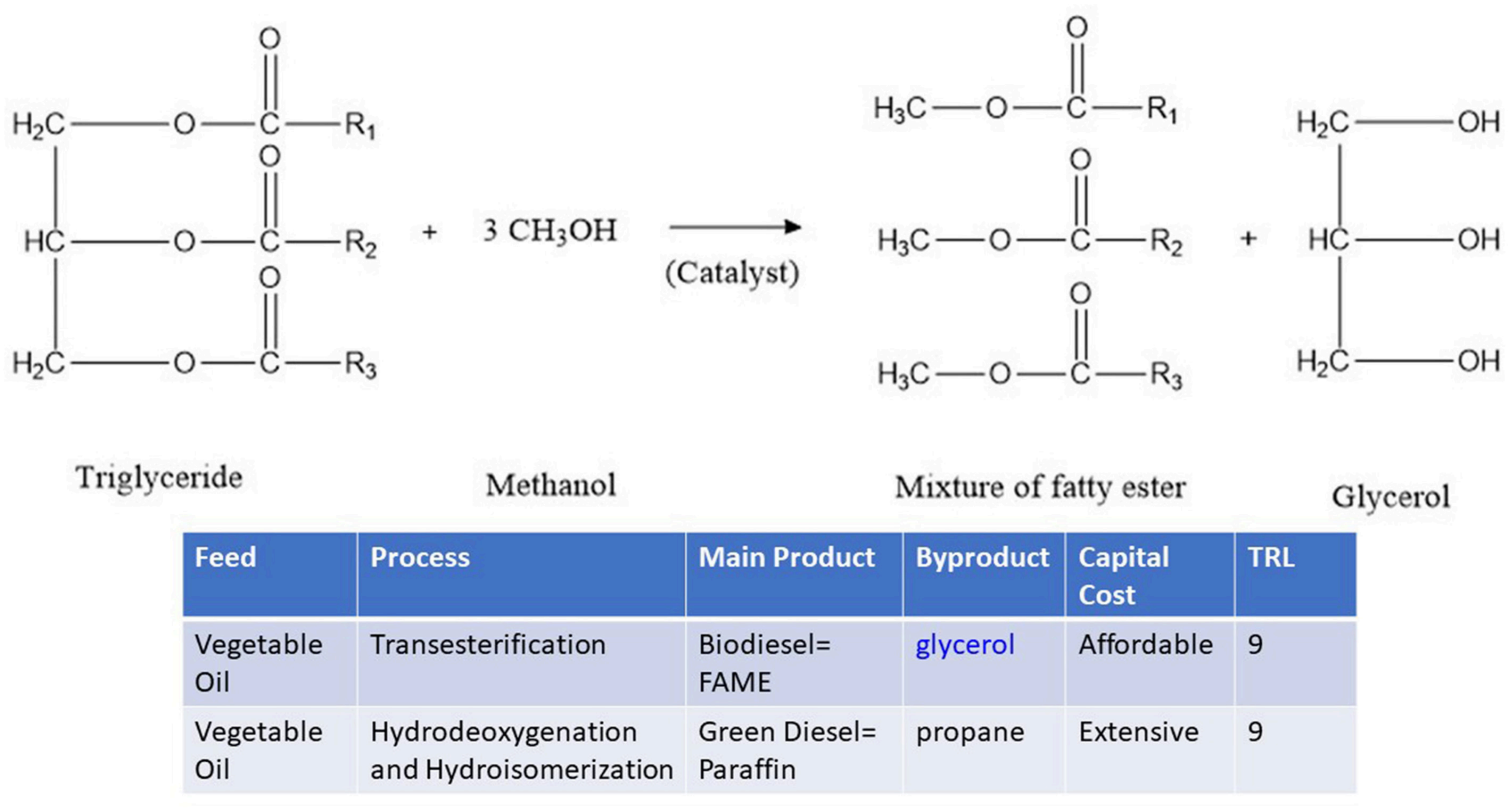
Biodiesel (FAMA) and glycerol from triglyceride of vegetable oil. TRL = technology readiness level.

The worldwide production of biodiesel is continuously increasing due to the growth and demand for transportation fuels and industry. In 2017, more than 30 billion liters of biodiesel (FAME) were produced. Correspondingly, ~6 billion liters of glycerol was produced (Table 1). Recently, many countries released higher blend mandates (Ren21, 2018). For instance, Indonesia recently released regulation for B20 (Biodiesel 20%). Thailand is targeting to use 9 million liters of biodiesel by 2020. This trend in biodiesel certainly will increase the production of glycerol, as a by-product in biodiesel plant. Glycerol (also known as glycerin, propane-1,2,3-triol, C_3_H_8_O_3_) is produced with a ratio of 1:1 with the biodiesel main product (Figure 1). The worldwide production of glycerol increased five-fold from 2006 to 2018 to reach 36 billion liters (Quispe et al., 2013; Monteiro et al., 2018). The abundance of glycerol as renewable chemicals should be monetized to improve the economic feasibility of biodiesel industry. Different pathways to convert glycerol to chemicals and fuels depend on the progress of low-cost and affordable catalysts (Atabani et al., 2012; Zakaria et al., 2012; Bagheri et al., 2015; Galadima and Muraza, 2016a,b; Monteiro et al., 2018).

**Table 1:** Worldwide production of biodiesel and glycerol in 2017 (Ren21, 2018).

**Country**	**Biodiesel** **(FAME)**	**Glycerol**	**HVO/Green Diesel**	**Blend mandate**
Unit	[billion liters]	[billion liters]	[billion liters]	% Biodiesel
USA	6.0	1.2	1.7	2 to 20
Brazil	4.3	0.86		8
Germany	3.5	0.7		
Argentina	3.3	0.66		10
China	1.0	0.2		
France	2.3	0.46		
Thailand	1.4	0.28		7 (9 million L by 2020)
Indonesia	2.5	0.5		20
Canada	0.5	0.1		2 to 4
the Netherlands	0.4	0.08	1.3	
Spain	1.3	0.26		11.3 (2020)
Poland	1.0	0.2		
Singapore		0	1.3	
India	0.2	0.04		15
Colombia	0.6	0.12		10
EU-28	11.8	2.36	3.5	
World	30.7	6.14	6.5	

*Density of glycerol at 20°C is 1.26 kg/l, while the density of biodiesel is ~ 0.99 kg/l. HVO is the abbreviation for hydrogenated vegetable oil*.

This mini-review paper aims to emphasize the potential exploration of zeolites for glycerol conversion to two promising commercial products: acrolein and aromatics with an emphasis on recent open literature after 2010. Many cited works are recent papers in the past 3 years from the 2016 to 2018 period.

**Figure 2:**
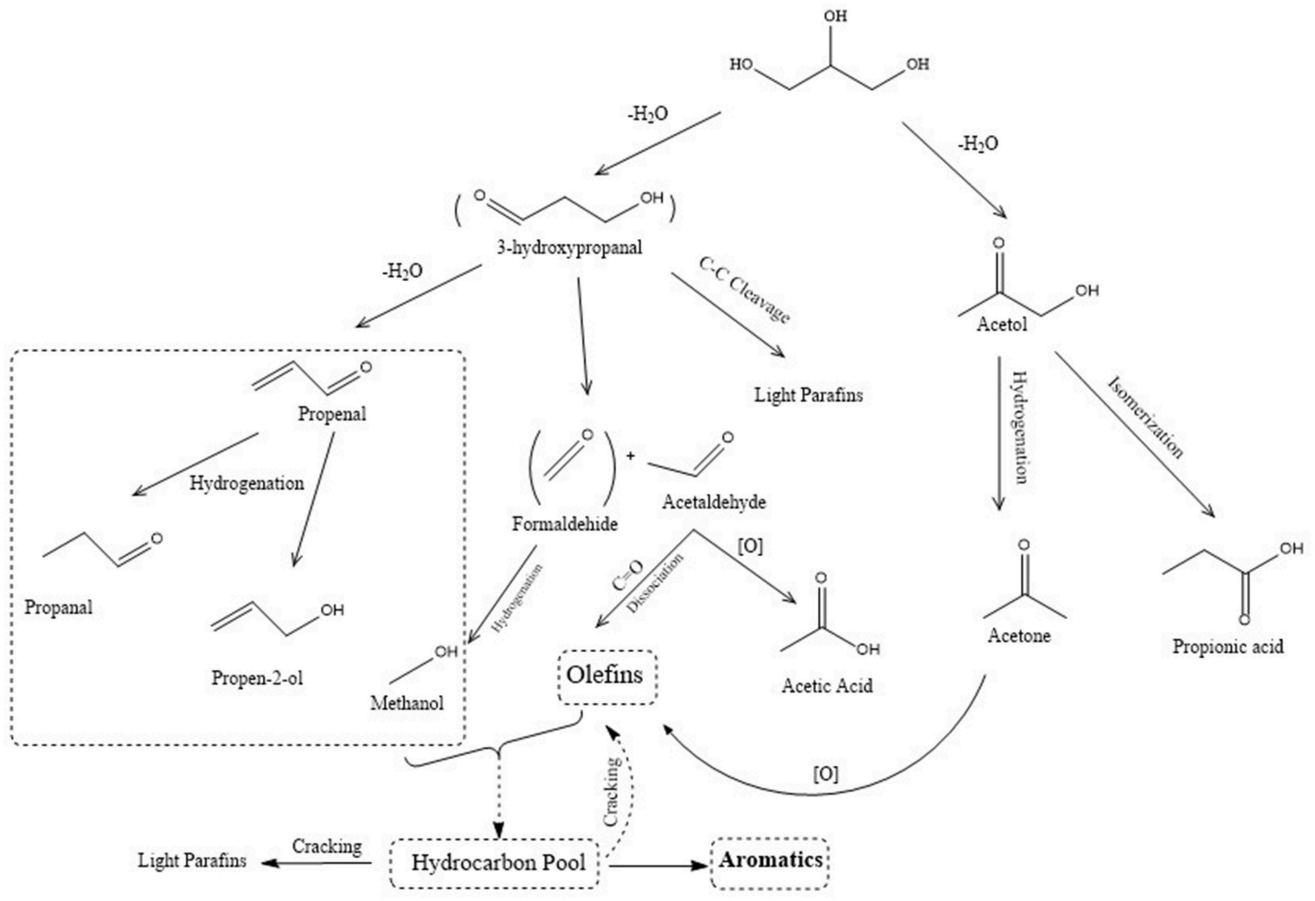
Scheme of glycerol to acrolein and aromatics (Tamiyakul et al., 2015).

The conversions of glycerol to fuels and value-added chemicals have been explored via different schemes such as acetalization, acetylation, dehydration, oxidation, and many others. The schemes can be classified into two main trends: (i) with co-reactant and (ii) without co-reactant. Different chemicals have been targeted from glycerol, among others; aromatics, acrolein, acetal, and glycerol carbonate were studied extensively over porous solid acid catalysts (Galadima and Muraza, 2016a,b; Mahdi et al., 2016) (see Figure 2). Table 2 presents the highlight of different conversion pathways from glycerol to chemicals over zeolite catalysts studied by different research groups recently. The explored co-reactants were acetone, n-butanal, benzyl alcohol, acetic acid, acetic anhydride, isobutylene, ketones, formaldehyde, acetaldehyde, benzaldehyde, and many others (Cornejo et al., 2017).

**Table 2:** Highlights on different pathways of glycerol conversions to chemicals over zeolites.

**Conversion**	**Catalyst**	**Results**	**References**
Glycerol conversion to aromatics via pyrolysis followed by aromatization	ZSM-5 (MFI) with SiO_2_/Al_2_O_3_ of 23. The matrix of bentonite was varied from 10, 20 and 40 wt.%.	Crude glycerol with 2.4 wt.% of H_2_O and 44.5 wt.% of free fatty acids was used as a feedstock. The pyrolysis (500°C) followed by aromatization (at 550°C). Conversion of glycerol = 100°C. The selectivity of aromatics (BTX) was 35% (Carbon) or 15 wt.%. S_Benzene_ = 27%, S_Toluene_ = 45 and S_Xylene_ = 28%. S_Benzene_ changed to 40% (additional 10%) when bentonite was added as matrix.	(He et al., 2018)
Glycerol to aromatics	H-MFI, Zn/MFI, 2.34 wt.% Sn MFI, Ni/MFI, Mo/MFI and Ag/MFI.	BTX aromatics (21.1 wt.%) with 10 h stability. Parent H-ZSM-5 only resulted 13.9 wt.% aromatics with 5.5 h stability.	(Wang et al., 2016, 2017, 2019)
Glycerol to acrolein	Zeolite Y (FAU) was modified by La and Pd-La.	The yield of acrolein increased from 57 to 75% at 300°C due to the increase of Brønsted and Lewis acidity.	(Gonzalez-Arellano et al., 2015)
Acetalization glycerol with acetone	Hierarchical zeolites from different topologies such as MFI, MOR and BEA.	High conversion (above 80%) and high selectivity to solketal (nearly 100%).	(Kowalska-Kus et al., 2017)
Glycerol to glycerol carbonate	Natural clinoptilolite (HEU) was dealuminated by 1 N of HCl for 90 min.	Reaction parameters were studied. The conversion of glycerol increased with a decrease of catalyst diameter.	(Mahdi et al., 2016)
Glycerol to solketal with acetone as a co-reactant	Zeolite Beta(Si/Al_2_ = 25) was compared with Y (FAU, Si/Al_2_ = 30) and MOR (Si/Al_2_ = 16).	Glycerol: acetone = 2:1. Beta was the most active (X_glycerol_ = ca.74) and the most selective to solketal (ca.98%). Nano BEA exhibited higher activity and higher selectivity to desired product.	(Manjunathan et al., 2015)
Glycerol to allyl alcohol	Hierarchical ZSM-5 was fabricated from commercial ZSM-5 (Si/Al = 40). Ag-ZSM-5 was prepared by IWI (incipient wetness impregnation).	Gas phase reaction, glycerol to allyl alcohol was studied. *P* = 0.1–4 MPa. Dehydration was followed by hydrogenation. Lewis acidity was reduced to increase acrolein selectivity. The 5 wt.% Ag-hierarchical ZSM-5 was the best catalyst with S _allylalcohol_ = 20%, X _glycerol_ = 80%. Stable for 100 h reaction.	(Manjunathan et al., 2015)
Glycerol etherification with n-butanol	H-BEA was compared with H-MFI.	Etherification of glycerol using n-butanol at 140–180°C and 0.5 MPa. X _glycerol_ = 55%. S _mono−butylglycerolether(MBGE)_ = 98%. MBGE is an additive to biodiesel.	(Nandiwale et al., 2014; Aghbashlo et al., 2019)
Glycerol etherification with benzyl alcohol	Starting material: NH_4_-ZSM-5 (MFI) with Si/Al = 40. Hierarchical MFI was prepared by NaOH desilication and HCl dealumination.	Higher acid sites were not linear with glycerol conversion. Three different acid sites resulted almost the same glycerol conversion in the range of 70 to 77%. Selectivity to bulky product di-benzyl-glycerol ether (DBG, 1,3-dibenzyloxy-2-propanol plus 1,2-dibenzyloxy-3-propanol) was higher for hierarchical MFI. Catalyst with higher Al content (Si/Al = ca 0.20) was more selective toward mono-benzyl-glycerol ether (MBG).	(Gonzalez-Arellano et al., 2015)

The valorization of glycerol to value-added products by addition of co-reactant have been explored with ketones, aldehydes, or alcohols as co-reactants (Cornejo et al., 2017). Most of the cases, the higher the concentrations of co-reactants, the higher yield of the products in glycerol valorization (Marimuthu et al., 2018; Chen et al., 2019). Unfortunately, the higher ratio on co-reactant to glycerol, the higher the exergy destruction rate (Gutiérrez Ortiz et al., 2012a,b; Hajjaji et al., 2014; Antonova et al., 2015; Aghbashlo et al., 2018a,b, 2019; Gholami et al., 2018; Presciutti et al., 2018).

Without the addition of co-reactant, acrolein and aromatics are among the most studied chemical products over zeolite catalysts (Galadima and Muraza, 2016a,b). Numerous catalytic materials were evaluated for glycerol to acrolein such as oxides, heteropoly acids, zeolites, and silicoaluminates (Galadima and Muraza, 2016a). There are many reviews on the conversions of glycerol to acrolein and aromatics. This paper focused only on zeolite catalysts for dehydration and aromatization.

## Glycerol-to-aromatics Over Zeolite Catalysts

Most of research groups reported that Brønsted acidity played important roles for the conversion of glycerol to aromatics over zeolite catalysts. Table 3 summarizes the conversion of glycerol to aromatics reported recently. Zeolites with MFI framework are the most studied material for the conversion of glycerol and methanol to aromatics. Yang and coworkers incorporated tin (Sn) into the framework of MFI with Si/Al_2_ of 200 (Yang et al., 2018). The Si/Al ratio plays critical role to tune the yield of aromatics (BTX). Higher Al content (low Si/Al) led to higher yield of aromatics (He et al., 2018). The addition of bentonite as a matrix increased the selectivity to benzene. However, coke deposition was significant and reduced the Brønsted acidity. The coke was removed in regeneration step at 600°C. During regeneration, some of the bentonite structures collapsed and the acid strength was reduced.

**Table 3:** Glycerol-to-aromatics over zeolite catalysts.

**Catalyst**	**Condition**	**C_**glycerol**_ (%)**	**S_**aromatics**_ (%)**	**Remark**	**References**
Dealuminated H-ZSM-5 (MFI) with initial Si/Al = 25	*T* = 400°C, glycerol/methanol = 40 wt.%, *P* = 0.1 MPa, WHSV: 0.71 h^−1^.	ca.98	ca.32 (C% BTX)	The most stable catalysts was the hierarchical ZSM-5 made by steam + acid dealumination = 11.5 h.	(Wang et al., 2019)
ZSM-5 (SiO_2_/Al_2_O_3_ = 30)	*T* = 400°C. WHSV = 0.8 h^−1^.	100	>30 (C% BTX)	In the presence of water as a contaminant, the catalyst was deactivated rapidly.	(Jang et al., 2014)
H-ZSM-5 (Si/Al_2_ = 200)	*T* = 400°C WHSV = 0.9 h^−1^.	100	18	Only 3 h life-time.	(Yang et al., 2018)
Hierarchical Sn-ZSM-5	*T* = 400 °C WHSV = 0.9 h^−1^.	100	32 (BTX)	H-Sn-ZSM-5 was desilicated by 0.3 M NaOH. Longer catalyst lifetime.	(Yang et al., 2018)
Pd-H-ZSM-5	*T* = 400°C. H_2_/glycerol = 10:1. P = 1 atm.	100	More than 50	Without Pd, the main product was acrolein with 11% aromatics. Pd was responsible for hydrodeoxygenation while H-ZSM-5 was for aromatization.	(Xiao and Varma, 2016)
Hierarchical ZSM-5. Starting material: ZSM-5 with Si/Al of 25. Different alkaline solutions were used for desilication.	*T* = 400°C. *P* = 1 atm. Feed = 40 wt.% glycerol in methanol. WHSV = 0.71 h^−1^.	100	15 wt.%	Desilication induced the increase of Brønsted acidity. The BTW increased while heavier aromatics ere suppressed.	(Wang et al., 2017)
H-Y (FAU), Si/Al = 40	*T* = 400°C. *P* = 2 MPa. W/F = 0.5 h. H_2_/glycerol = 15:1.	ca. 95	31	FAU was less selective to aromatics, as compared to MFI.	(Hoang et al., 2010)
H-ZSM-5 (MFI) with Si/Al = 45	*T* = 400°C. *P* = 2 MPa. W/F = 0.5 h. H_2_/glycerol = 15:1.	ca. 95	59	Maximum yield of aromatics was ca. 60%.	(Hoang et al., 2010)

*Glycerol to the second reactant ratio was presented as molar ratio. All conversions and selectivity values were rounded to the nearest whole number*.

The effect of metal (M) modified zeolite, especially M-ZSM-5 was reported recently (Xiao and Varma, 2016). Without palladium (Pd), the main product was acrolein (54%) with 11% of aromatics. The Pd was responsible for hydrodeoxygenation (HDO) while H-ZSM-5 was in charge for aromatization. Pd was obviously more active than platinum (Pt) in hydrogenation. The metal improved the scission of C-O bond. The temperature was crucial to increase the conversion of glycerol. The optimum temperature was found to be ca. 400°C. The pressure has a negligible effect on the conversion of glycerol to aromatics.

Hierarchical ZSM-5 (MFI), fabricated by nitric acid dealumination and steam-treatment at 500°C, exhibited stable conversion of glycerol (ca.98%) to aromatics (ca. 32% - C-based BTX) (Wang et al., 2019). The strong acidity on the surface of ZSM-5 affected the stability negatively, the catalyst life-time for conventional H-ZSM-5 (Si/Al = 25) was very short. Strong acidity favored the formation of hydrocarbon pool species such as polymethylbenzenes inside the MFI intersection and resulted in more coke deposition. The H-ZSM-5 contains sinusoidal channels (0.51 × 0.5 nm), which are crossed with straight channels (0.53 x 0.56 nm) with intersection channel of 0.9 nm size (Corma et al., 2008; Lauriol-Garbay et al., 2011).

The conversion of glycerol to aromatics was increased by the addition of appropriate amount of water to the system at 440°C under atmospheric pressure as reported by Jang and coworkers in 2014 (Jang et al., 2014). The maximum aromatic products were obtained approximately with 25 wt.% of glycerol in glycerol-water mixture. Mesoporosity has an important role to control the selectivity to aromatics (Wang et al., 2017). Hierarchical ZSM-5, fabricated by desilication with initial Si/Al of 25 has higher Brønsted acidity than its parent. The increase of Brønsted acidity favored higher selectivity to aromatics (benzene-toluene-xylene) with suppressed production of heavier aromatics. Pore topology was also crucial to adjust the selectivity to aromatics. The large pore FAU zeolite (with pore size of 0.74 nm) was less selective to aromatics, as compared to MFI (with pore size of approximately 0.55 nm (Hoang et al., 2010).

## Glycerol-to-acrolein Over Zeolites

**Figure 3:**
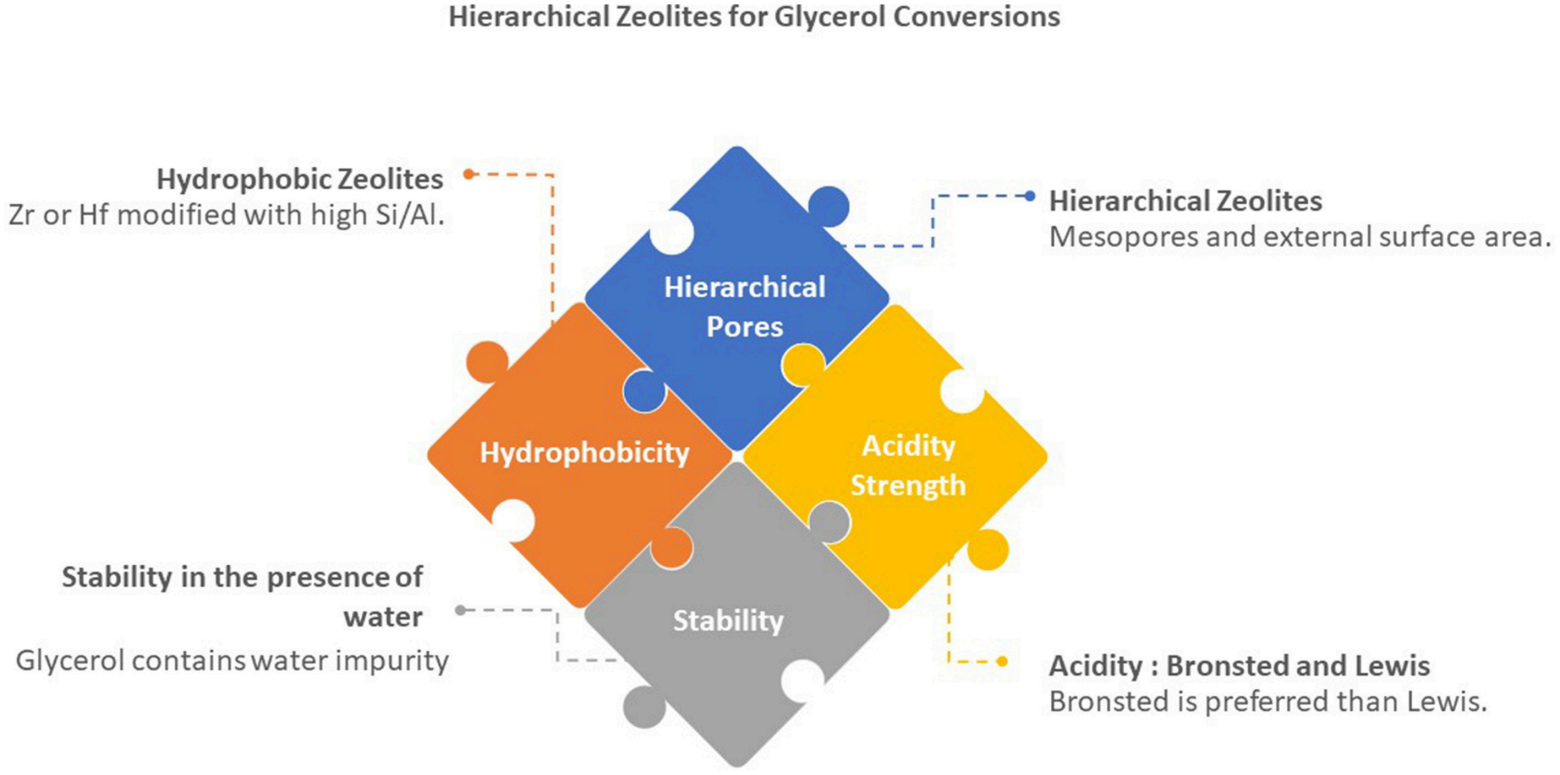
Crucial factors in the applications of hierarchical zeolites in glycerol conversions.

The catalytic activity of zeolites in glycerol to acrolein was determined by some strong factors: (i) shape selectivity of medium pore zeolites, (easy access of active sites in hierarchical zeolites (i) the number of acid sites, (ii) the presence of mesopores, (iii), and (iv) the hydrophobicity of the catalyst (as described in Figure 3). Table 4 presents selected works on glycerol to acrolein over zeolites.

**Table 4:** Selected works on **glycerol-to-acrolein** over zeolites.

**Catalyst**	**Condition**	**X_**glycerol**_ (%)**	**S_**acrolein**_ (%)**	**Remark**	**References**
Hierarchical and conventional SAPO-40 (AFR)	*T* = 320 °C. *P* = 1 atm. WHSV: 0.85 h^−1^.	100	78	Hierarchical AFR was more active, more selective to acrolein. AFR was also more active as compared to SAPO-11 and SAPO-34.	(Fernandes et al., 2017)
MCM-22 (MWW) with Si/Al2 = 28	Feed: glycerol in water (36.6%), *T* = 320°C. *T* = 2 h.	99.8	~50	Coke after 10 h = ca. 25%.	(Carriço et al., 2016)
Delaminated MCM-22 (ITQ-2, MWW) with Si/Al2 = 37	Feed: glycerol in water (36.6%), *T* = 320°C. *T* = 2 h.	58	44	Delaminated MWW, ITQ-2 has the largest V_meso_.	(Carriço et al., 2016)
Pillared MWW (MCM-36), Si/Al2 = 51)	Feed: glycerol in water (36.6%), *T* = 320°C. *T* = 2 h.	89	ca.7	MCM-36 has B/L = 0.9.	(Carriço et al., 2016)
H-Na-mordenite (MOR), Si/Al = 8	*T* = 400°C. *P* = 2 MPa. W/F = 0.5 h. H_2_/glycerol = 15:1.	ca.93	27	MOR, is considered as 1D pore zeolite with larger size (12 MR, 0.65 x 0.7 nm) than TON zeolite.	(Hoang et al., 2010)
H-ZSM-22 (TON), Si/Al = 45	*T* = 400°C. *P* = 2 MPa. W/F = 0.5 h. H_2_/glycerol = 15:1.	100	ca. 80	TON is representing 1D pore zeolite with medium pore size (10 MR, 0.46 x 0.57 nm).	(Hoang et al., 2010)
ZSM-5 (SiO_2_/Al_2_O_3_ = 30)	*T* = 400°C. WHSV = 0.8 h^−1^.	100	>30 (C% BTX)	In the presence of water as a contaminant, the catalyst was deactivated rapidly.	(Jang et al., 2014)

*Glycerol to the second reactant ratio was presented as molar ratio. **C**_**glycerol**_ = Conversion of glycerol. **S**_**acrolein**_ = selectivity to acrolein*.

Carrico et al. reported the utilization of MWW, pillared MWW and delaminated MWW in glycerol to acrolein at 320°C (Carriço et al., 2016). The selectivity to acrolein increased with the increase of acid site density. ITQ-2, with the strongest of density of acid sites exhibited the best selective solid for acrolein. The mesopores were not linear with acrolein selectivity. More coke depositions were observed over ITQ-2 which had the largest mesopore pore volumes. The Brønsted/Lewis (B/L) ratio is a crucial parameter to maximize acrolein selectivity. The higher the B/L has the higher selectivity to acrolein. The low B/L was important if we need to maximize acetol (hydroxyacetone).

## Glycerol Conversion Over Natural Minerals

Glycerol is an abundant and low-cost feedstock. To minimize the barrier in the valorization of glycerol, low-cost catalysts are targeted. Despite of their low-cost benefit, there are still limited works on the use of natural zeolites and natural clays for glycerol conversions to chemicals. Some notable works are cited in Table 5. The most common natural zeolites in solid-acid catalysts are from HEU frameworks. Mahdi et al reported clinoptilolite (HEU) in glycerol conversion to glycerol carbonate (Mahdi et al., 2016). Furthermore, recently our group also reported natural mordenite and hierarchical mordenites (Nasser et al., 2016b; Kurniawan et al., 2017a,b). Mild activation of natural zeolites and clays should be established to achieve industrial standards for safety and economic assessments (Pawar et al., 2015).

**Table 5:** Glycerol conversion over natural zeolites and natural minerals.

**Co-reactant**	**Catalyst**	**T (°C)**	**X _**glycerol**_ (%)**	**Products**	**Remark**	**References**
No co-reactant	Bentonite was added to ZSM-5 with different weight ratios: 10, 20 and 40 wt.%.	500°C (pyrolysis) or 550°C (aromatization).	100	aromatics BTX, 35%.	Bentonite was selective to benzene. S_Benzene_ changed to 40% (additional 10%) when bentonite was added as matrix.	(He et al., 2018)
Benzaldehyde	montmorillonite	40	83%	Solketal (99%)	Glycerol:benzaldehyde dimethyl acetal = 1:1.1 for 6 h.	(Nanda et al., 2016)
Sodium bicarbonate	Hierarchical clinoptilolite	60–100	28	N.A.	The optimum conditions at molar ratio of glycerol: sodium bicarbonate: water equal to 3:1:3.	(Mahdi et al., 2016)
Acetone	montmorillonite	30	87%	Solketal (85%)	Glycerol:acetone = 1:6, catalyst weight: 3 wt.% of total reactant weight, for 2 h.	(Sandesh et al., 2015)

*Glycerol to co-reactant ratio was highlighted as molar ratio*.

In addition to activation procedures, stability during chemical reactions, and possible regeneration are expected to suppress the catalyst cost. Ahmed and coworkers reported the decrease of crystallinity of hierarchical MRE zeolite (i.e., ZSM-48) (Ahmed et al., 2017). Typically, the crystallinity of zeolite-based catalysts decreased during regeneration. Before the hierarchical zeolites can be applied in glycerol conversions, improvements on the stability of structure and acidity after regeneration should be established.

The additions of boron and fluoride have been explored to improve the stability of zeolite catalysts in the presence of hot water or steam (Sanhoob et al., 2017). Boron has a weaker acidity as compared to aluminum acid sites. Appropriate level of boron content increased the stability of MFI framework (with zero aluminum) in steam-assisted reactions. The conversion in the presence of steam was maintained with the large external surface area with higher acid strength and high concentration of silanols. Proper compositions of minerals were crucial.

Considering the deactivation problems in glycerol conversion over zeolite catalysts, several cheaper catalysts are proposed: (i) natural zeolites which need acid-base activation, (ii) OSDA-free zeolites, (iii) amine-templated zeolites. Those materials were considered due to cheaper production cost. OSDA-free zeolites are synthesized without template (without organic structure directing agent). Amines are low-cost OSDA as compared to ammonium hydroxide OSDAs.

## Perspective on Glycerol Over Hierarchical Zeolites in the Presence of Water

The crude glycerol obtained as byproduct in biodiesel production contains water (up to 10%), ash, matter organic non-glycerol (MONG) and trimethylene glycol (Tan et al., 2013). The presence of water, hot water or steam in glycerol conversions affects the stability of zeolites (including hierarchical zeolites). Table 6 presents some notable research reports on the effect of water in glycerol conversions. High Si/Al (Si/Al = 16) zeolite beta (BEA) with rich silicon content has resulted in the hydrophobic properties for the conversion of glycerol with formaldehyde in the presence of water (Da Silva et al., 2009). This may happen over BEA as this topology has the internal surface area almost similar with the external surface area. The authors claimed that the water molecules were prevented to reach the acid sites at the internal pore architectures. Surprisingly, zeolite FAU (USY) exhibited negligible conversion due to hydrophilic properties of FAU (Si/Al = 28). Good stability was also observed over medium pore zeolite, such as MFI with Si/Al of 14 with the pore opening of ca. 0.56 nm, the formation glycerol acetals was prevented.

**Table 6:** Glycerol conversions over zeolites in the presence of water.

**Co-reactant**	**Catalyst**	**T (°C)**	**X_**glycerol**_ (%)**	**Selectivity to main product**	**Remark**	**References**
No co-reactant, but different solvents were used.	ZSM-5 (MFI) with Si/Al_2_ of 30.	440	>99.99	The carbon yield of aromatics reached 25 wt.% after ca. 5 h.	Different concentrations of water were used. Jang et al reported the positive effect of water in glycerol feed. The glycerol/(glycerol+water) was varied from 30 to 70 wt.%.	(Jang et al., 2014)
No co-reactant	MCM-22 (MWW) with Si/Al_2_ = 28	320	99.8	S _acrolein_ = ~50. Coke after 10 h = ca. 25%.	Feed: glycerol in water (36.6%), *t* = 2 h.	(Carriço et al., 2016)
No co-reactant	Delaminated MCM-22 (ITQ-2, MWW) with Si/Al_2_ = 37	320	58	S _acrolein_ = 44. Delaminated MWW, ITQ-2 has the largest Vmeso.	Feed: glycerol in water (36.6%), *t* = 2 h.	(Carriço et al., 2016)
No co-reactant	SAPO-40 (AFR) with 12 MR (0.67 nm).	320	89	S _acrolein_ = 72%.	*P* = 1 atm. WHSV: 0.85 h-1. *T* = 2.5 h. Feed: 10 wt.%.	(Fernandes et al., 2017)
No co-reactant	Hierarchical SAPO-40 (AFR)	320	100	S _acrolein_ = 78%.	Hierarchical SAPO-40 was more active and more stable.	(Fernandes et al., 2017)
Acetone, 20 wt.% of water	Zeolite Y (FAU), Si/Al = 2.6	70	36	Selectivity was not clearly reported.	Conversion in the presence of water decreased to 19% where glycerol/H_2_O: 4:1.	(Li et al., 2012)
Formaldehyde and water with formaldehyde (37%)	Zeolite BEA with Si/Al = 16	70	95 after 60 min (0.5 h)	70% of 6 MR (membered rings) acetals and 30% of 5 MR.	Glycerol: formadehyde = 1: 1.2	(Da Silva et al., 2009)
No co-reactant	ZSM-5 (MFI) with Si/Al = 50, Particle size: 40–120 micron.	350	97	S _acrolein_ = 59%.	Feed: 85 wt.% glycerol in the presence of water.	(Corma et al., 2008)
No co-reactant	ZSM-5 (MFI) with Si/Al = 50. Particle size: 40–120 micron.	350	100	S _acrolein_ = 59%.	Feed: 50 wt.% glycerol in the presence of water.	(Corma et al., 2008)
No co-reactant	ZSM-5 (MFI) with Si/Al = 50. Particle size: 40–120 micron.	350	100	S _acrolein_ = 62%.	Feed: 20 wt.% glycerol in the presence of water.	(Corma et al., 2008)
No co-reactant	Hierarchical ZSM-5 with similar Si/Al	250–320	100	S _acrolein_ = 81%.	Selectivity to acrolein was relatively constant around 80%.	(Zhang et al., 2015)
No co-reactant	Hierarchical ZSM-5, intercrystalline mesopores	250–320	100	S _acrolein_ = 86%.	Hierarchical ZSM-5 with intercrystalline mesopores has exhibited the highest selectivity to acrolein.	(Zhang et al., 2015)
No co-reactant	Hierarchical ZSM-5, intracrystalline mesopores	250–320	100	S _acrolein_ = 85%.	Hierarchical ZSM-5 with intracrystalline mesopores showed lower stability than the hierarchical ZSM-5 with intracrystalline mesopores.	(Zhang et al., 2015)

*X_glycerol_ (%) = conversion of glycerol*.

Bakare and co-workers reported that a one dimensional pore zeolite, MTT framework was stable in the presence of water vapor at 200°C under autogenous pressure of autoclave (Bakare et al., 2016). The addition of lanthanum (La) and cerium (Ce) improved the stability of MTT in hot water vapor. The most stable catalyst was cerium modified MTT. Stability of TON, another medium pore zeolite with one-dimensional pore architectures was reported by (Jamil et al., 2016).

Li et al. (2012) reported the presence of water reduced the catalytic activity of solid acid catalysts (up to 50%) in the acid-catalyzed glycerol conversions as compared with the activity with pure glycerol feed. They emphasized that strength and density of Brønsted and Lewis do not have linear relationship with catalyst activity. Hierarchical large pore zeolite (FAU) and mesoporous silica (Al-TUD-1) with high number Brønsted and Lewis acidity were not active enough in the conversion of glycerol to solketal, a fuel additive (Li et al., 2012). Hydrophobicity of the catalysts plays an important role in solid-acid catalyzed reactions. In line with hydrophobicity strategy, the addition of zirconium and hafnium improved the stability of aluminosilicate TUD-1 and SBA-15 in glycerol conversions (Ammaji et al., 2018).

Prinsen and co-workers reported the activity of the solid acid catalyst is not always linear increase with acidity for the conversions of glycerol and fatty acids to chemicals (Prinsen et al., 2018). In the conversion of palmitic acid to methyl palmitate over zeolites, it was reported that zeolite Y (FAU) with high a Si/Al of 30 (SiO_2_/Al_2_O_3_ = 60) was more active than Al-rich FAU (with SiO_2_/Al_2_O_3_ of 5. FAU based catalyst was also more active as compared with medium pore ZSM-5 (MFI). Maximum 100% conversion was achieved at the temperature reaction of 70°C for 3 h. They concluded that the most important parameters were: (i) porosity of zeolites (hierarchical pores), (ii) hydrophobicity, (iii) acid strength and (iv) stability as shown in Figure 3. The importance of hierarchical pores was also reported by Zhang et al. (2015). Hydrophobicity of catalysts are tunable by adding metals such as zirconium and hafnium and by removing the aluminum from the zeolite frameworks.

The effects of types of mesopores, either intercrystalline mesopores or intracrystalline mesopores were reported by Zhang et al. (2015). The most suitable nanozeolites with intercrystalline mesopores followed by hierarchical ZSM-5 with intra crystalline mesopores. Selectivity to acrolein was slightly increased due to intrercrystalline and intracrystalline mesopores. Fernandes et al. (2017) reported the application of hierarchical SAPO-40 (AFR) zeolite for the conversion of glycerol to acrolein in the presence of high water content (10 wt.% of glycerol in water). The hierarchical SAPO-40 with higher mesopore volume promoted larger stability and less mass-transfer limitation. The hierarchical SAPO-40 contained less Brønsted acidity with almost similar Lewis acid strength. The unique SAPO-40 was synthesized using two different silica sources, including [3-(trimethoxysilyl) propyl] octadecyl dimethyl ammonium chloride (TPOAC) and fumed silica. In the conversion of glycerol to acrolein, SAPO-40 was more active than SAPO-11 (AEL) and SAPO-34 (CHA). Due to more open porosity, the coke found in hierarchical AFR was higher (23.5%). The presence water (10 wt.% glycerol) did not affect the stability of SAPO-40 negatively. Corma et al. (2008) reported interested fact about glycerol conversion that the addition of water increased the selectivity to acrolein over commercial zeolite Y in SiO_2_-Al_2_O_3_ matrix and ZSM-5 in clay matrix.

The improvement of catalytic activity of zeolites in glycerol dehydration and aromatization by the creation of mesopores are reported in Table 5. Nanosized zeolites are also promising for glycerol conversions. The nanosized crystals can also form intercrystalline hierarchical zeolites (Kowalska-Kus et al., 2017; Possato et al., 2017; Feliczak-Guzik, 2018; Galadima and Muraza, 2018; Yang et al., 2018; Ahmed et al., 2019). Theoretically, the intercrystalline zeolites affect better diffusion properties to tackle mass-transfer limitations (Yu et al., 2013; Manjunathan et al., 2015; Huang et al., 2017). Hierarchical zeolites are fabricated by different approaches, however, to suppress catalyst cost (as part of variable cost in industrial scale), the templated methods are not proposed due to the expensive price of the bulky organic structure directing agents (OSDA). The low-cost desilication and dealumination routes are preferred. The demetalization processes changed the mesopore distribution, improved the access to active sites, reduced mass transfer limitation and acid distributions. Another route for cheap catalyst is OSDA free (without additional OSDA). The low-cost fabrication of hierarchical zeolites with different topologies were started with three-dimensional (3D) medium pore ZSM-5 (MFI) (Muraza et al., 2013, 2014; Bawah et al., 2018), 3D large Beta (BEA) and 3D large pore FAU. Later, some one-dimensional pore zeolites such as MTW (Sanhoob et al., 2016), MTT (Muraza et al., 2013, 2014), TON (Jamil et al., 2018), and MRE (Ahmed et al., 2017) were also reported elsewhere.

Considering the popularity and the stability of hierarchical zeolites for glycerol monetization in water, hot water, sub-critical water and super critical water, it is also beneficial to explore the applicability of low-cost zeolites with hierarchical pores. The manufacturing of zeolites is indeed possible by using organic-structure directing agent (OSDA) free synthesis. The absence of template or OSDA (Khalil and Muraza, 2016; Idris et al., 2019; Nasser et al., 2019) will suppress the catalyst cost as one of the variable costs in glycerol biorefinery. Pure MOR, for instance, was fabricated by using OSDA or without OSDA route. The synthesis parameters such as alkalinity (Na/Si) and Si/Al were found to be crucial to obtain pure mordenite (Idris et al., 2019). If higher Si/Al is targeted, the higher Na/Si ratio should be set in the synthesis mixture. Rapid fabrication of low-cost MOR zeolite was also possible under microwave irradiation (Khalil and Muraza, 2016).

In addition, the natural zeolites are also potential as a low-cost catalyst for glycerol conversions. Mostly, natural zeolites are presence as small pore zeolites such as clinoptilolite (HEU) and chabazite (CHA) (Aysan et al., 2016). There are more than 28 different zeolite frameworks found in nature (Stocker et al., 2017), mordenite (MOR) is a promising natural zeolites for industrial catalytic applications. Recently, Kurniawan and co-workers reported to the applications of MOR and hierarchical MOR from natural zeolites for catalytic reactions (Nasser et al., 2016a,b; Kurniawan et al., 2017b). Hierarchical mordenite was modified by zirconium and applied in glycerol conversion (i.e., esterification) (Popova et al., 2017). By this approach, natural zeolites can also be converted to hierarchical MOR as low-cost catalyst for glycerol conversions. Other medium pore natural zeolites are expected. In addition to natural zeolites, low-cost natural clays such as montmorillonite are expected to be applicable in the conversion of low-cost feedstock like glycerol. Montmorillonite, which was activated by sulfuric acid, was used as a catalyst support (Samudrala et al., 2018). Bentonite was reported as good matrix for ZSM-5 for glycerol conversions to aromatics (He et al., 2018). Bentonite contains approximately 80 wt.% of montmorillonite structured clay (Carniato et al., 2018; Pentrák et al., 2018).

## Perspective and Conclusions

This mini review highlights recent development glycerol conversions to acrolein and aromatics over zeolite-based catalysts. Crude glycerol usually contains water and other impurities. Water is a critical factor for glycerol conversions over solid acid catalysts. The presence of water and coke formation affect the stability of zeolite catalysts in glycerol conversions. Therefore, it was proposed to consider some strategies to suppress catalyst costs by using OSDA-free zeolites or by applying natural zeolites. Proper activations of natural zeolites were proposed. Natural mordenite is a potential natural zeolite for glycerol conversions. Low-cost catalysts should be found for low cost feedstock (e.g. glycerol). Coking is one of the main problems in the conversion of glycerol to acrolein. Coke formation can be suppressed by selecting the appropriate pore opening, especially medium pore zeolites with mild acidity. In addition to pore architectures of the catalysts, acidity is an important factor in glycerol to acrolein or aromatics over zeolite catalysts. This review will open plethora in designing better zeolite catalysts for glycerol conversions to acrolein and aromatics. Medium pore zeolite with shape selective catalysts are expected to perform better in glycerol to acrolein. Large pore zeolite with large cavity are expected to be ideal catalysts for glycerol to aromatics. Natural clays such as montmorillonite and natural zeolites are potential applied in commercial glycerol to aromatics.

## Author Contributions

The author confirms being the sole contributor of this work and has approved it for publication.

### Conflict of Interest Statement

The author declares that the research was conducted in the absence of any commercial or financial relationships that could be construed as a potential conflict of interest.

## References

[B1] Aghbashlo M. Hosseinpour S. Tabatabaei M. Rastegari H. Ghaziaskar H. S. (2019). Multi-objective exergoeconomic and exergoenvironmental optimization of continuous synthesis of solketal through glycerol ketalization with acetone in the presence of ethanol as co-solvent. Renew. Energy 130, 735–748. 10.1016/j.renene.2018.06.103

[B2] Aghbashlo M. Tabatabaei M. Hosseinpour S. Rastegari H. Ghaziaskar H. S. (2018a). Multi-objective exergy-based optimization of continuous glycerol ketalization to synthesize solketal as a biodiesel additive in subcritical acetone. Energy Conver. Manage. 160, 251–261. 10.1016/j.enconman.2018.01.044

[B3] Aghbashlo M. Tabatabaei M. Rastegari H. Ghaziaskar H. S. Roodbar Shojaei T. (2018b). On the exergetic optimization of solketalacetin synthesis as a green fuel additive through ketalization of glycerol-derived monoacetin with acetone. Renew. Energy 126, 242–253. 10.1016/j.renene.2018.03.047

[B4] Ahmed M. H. M. Muraza O. Galadima A. Yoshioka M. Yamani Z. H. Yokoi T. (2019). Choreographing boron-aluminum acidity and hierarchical porosity in ^*^BEA zeolite by in-situ hydrothermal synthesis for a highly selective methanol to propylene catalyst. Micropor. Mesopor. Mater. 273, 249–255. 10.1016/j.micromeso.2018.06.036

[B5] Ahmed M. H. M. Muraza O. Nakaoka S. Jamil A. K. Mayoral A. Sebastian V. (2017). Stability assessment of regenerated hierarchical ZSM-48 zeolite designed by post-synthesis treatment for catalytic cracking of light naphtha. Energy Fuels 31, 14097–14103. 10.1021/acs.energyfuels.7b02796

[B6] Ammaji S. Rao G. S. Chary K. V. R. (2018). Acetalization of glycerol with acetone over various metal-modified SBA-15 catalysts. Appl. Petrochem. Res. 8, 107–118. 10.1007/s13203-018-0197-6

[B7] Antonova Z. A. Krouk V. S. Pilyuk Y. E. Maksimuk Y. V. Karpushenkava L. S. Krivova M. G. (2015). Exergy analysis of canola-based biodiesel production in Belarus. Fuel Proc. Technol. 138, 397–403. 10.1016/j.fuproc.2015.05.005

[B8] Atabani A. E. Silitonga A. S. Badruddin I. A. Mahlia T. M. I. Masjuki H. H. Mekhilef S. (2012). A comprehensive review on biodiesel as an alternative energy resource and its characteristics. Renew. Sustain. Energy Rev. 16, 2070–2093. 10.1016/j.rser.2012.01.003

[B9] Aysan H. Edebali S. Ozdemir C. CeliK Karakaya M. Karakaya N. (2016). Use of chabazite, a naturally abundant zeolite, for the investigation of the adsorption kinetics and mechanism of methylene blue dye. Micropor. Mesopor. Mater. 235, 78–86. 10.1016/j.micromeso.2016.08.007

[B10] Bagheri S. Julkapli N. M. Yehye W. A. (2015). Catalytic conversion of biodiesel derived raw glycerol to value added products. Renew. Sustain. Energy Rev. 41, 113–127. 10.1016/j.rser.2014.08.031

[B11] Bakare I. A. Muraza O. Kurniawan T. Yamani Z. H. Shafei E. N. Punetha A. K. (2016). Hydrothermal stability of MTT zeolite in hot water: the role of La and Ce. Micropor. Mesopor. Mater. 233, 93–101. 10.1016/j.micromeso.2015.11.038

[B12] Bawah A.-R. Malaibari Z. O. Muraza O. (2018). Syngas production from CO2 reforming of methane over Ni supported on hierarchical silicalite-1 fabricated by microwave-assisted hydrothermal synthesis. Int. J. Hydrog. Energy 43, 13177–13189. 10.1016/j.ijhydene.2018.05.073

[B13] Carniato F. Bisio C. Evangelisti C. Psaro R. Dal Santo V. Costenaro D.. (2018). Iron-montmorillonite clays as active sorbents for the decontamination of hazardous chemical warfare agents. Dalton Trans. 47, 2939–2948. 10.1039/C7DT03859C 29441378

[B14] Carriço C. S. Cruz F. T. Dos Santos M. B. Oliveira D. S. Pastore H. O. Andrade H. M. C. (2016). MWW-type catalysts for gas phase glycerol dehydration to acrolein. J. Catal. 334, 34–41. 10.1016/j.jcat.2015.11.010

[B15] Chen Z. Gao L. Han W. Zhang L. (2019). Energy and exergy analyses of coal gasification with supercritical water and O2-H2O. Appl. Thermal Eng. 148, 57–63. 10.1016/j.applthermaleng.2018.10.050

[B16] Corma A. Huber G. W. Sauvanaud L. O'connor P. (2008). Biomass to chemicals: catalytic conversion of glycerol/water mixtures into acrolein, reaction network. J. Catal. 257, 163–171. 10.1016/j.jcat.2008.04.016

[B17] Cornejo A. Barrio I. Campoy M. Lázaro J. Navarrete B. (2017). Oxygenated fuel additives from glycerol valorization. main production pathways and effects on fuel properties and engine performance: a critical review. Renew. Sustain. Energy Rev. 79, 1400–1413. 10.1016/j.rser.2017.04.005

[B18] Da Silva C. X. A. Gonçalves V. L. C. Mota C. J. A. (2009). Water-tolerant zeolitecatalyst for the acetalisation of glycerol. Green Chem. 11, 38–41. 10.1039/B813564A

[B19] Exxonmobil (2017). 2018 Outlook for Energy: A View to 2040. Exxonmobil.

[B20] Feliczak-Guzik A. (2018). Hierarchical zeolites: synthesis and catalytic properties. Micropor. Mesopor. Mater. 259, 33–45. 10.1016/j.micromeso.2017.09.030

[B21] Fernandes A. Filipa Ribeiro M. Lourenço J. P. (2017). Gas-phase dehydration of glycerol over hierarchical silicoaluminophosphate SAPO-40. Catal. Commun. 95, 16–20. 10.1016/j.catcom.2017.02.015

[B22] Galadima A. Muraza O. (2016a). A review on glycerol valorization to acrolein over solid acid catalysts. J. Taiwan Inst. Chem. Eng. 67, 29–44. 10.1016/j.jtice.2016.07.019

[B23] Galadima A. Muraza O. (2016b). Sustainable production of glycerol carbonate from by-product in biodiesel plant. Waste Biomass Valorization 8, 141–152. 10.1007/s12649-016-9560-y

[B24] Galadima A. Muraza O. (2018). Hydrocracking catalysts based on hierarchical zeolites: a recent progress. J. Indust. Eng. Chem. 61, 265–280. 10.1016/j.jiec.2017.12.024

[B25] Gholami A. Hajinezhad A. Pourfayaz F. Ahmadi M. H. (2018). The effect of hydrodynamic and ultrasonic cavitation on biodiesel production: an exergy analysis approach. Energy 160, 478–489. 10.1016/j.energy.2018.07.008

[B26] Gonzalez-Arellano C. Grau-Atienza A. Serrano E. Romero A. A. Garcia-Martinez J. Luque R. (2015). The role of mesoporosity and Si/Al ratio in the catalytic etherification of glycerol with benzyl alcohol using ZSM-5 zeolites. J. Mol. Catal. A Chem. 406, 40–45. 10.1016/j.molcata.2015.05.011

[B27] Gutiérrez Ortiz F. J. Ollero P. Serrera A. Galera S. (2012a). An energy and exergy analysis of the supercritical water reforming of glycerol for power production. Int. J. Hydrogen Energy 37, 209–226. 10.1016/j.ijhydene.2011.09.058

[B28] Gutiérrez Ortiz F. J. Ollero P. Serrera A. Galera S. (2012b). Process integration and exergy analysis of the autothermal reforming of glycerol using supercritical water. Energy 42, 192–203. 10.1016/j.energy.2012.03.069

[B29] Hajjaji N. Baccar I. Pons M.-N. (2014). Energy and exergy analysis as tools for optimization of hydrogen production by glycerol autothermal reforming. Renew. Energy 71, 368–380. 10.1016/j.renene.2014.05.056

[B30] He S. Muizebelt I. Heeres A. Schenk N. J. Blees R. Heeres H. J. (2018). Catalytic pyrolysis of crude glycerol over shaped ZSM-5/bentonite catalysts for bio-BTX synthesis. Appl. Catal. B Environ. 235, 45–55. 10.1016/j.apcatb.2018.04.047

[B31] Hoang T. Q. Zhu X. Danuthai T. Lobban L. L. Resasco D. E. Mallinson R. G. (2010). Conversion of glycerol to alkyl-aromatics over zeolites. Energy Fuels 24, 3804–3809. 10.1021/ef100160y

[B32] Huang G. Ji P. Xu H. Jiang J.-G. Chen L. Wu P. (2017). Fast synthesis of hierarchical Beta zeolites with uniform nanocrystals from layered silicate precursor. Micropor. Mesopor. Mater. 248, 30–39. 10.1016/j.micromeso.2017.03.060

[B33] Idris A. Khalil U. Abdulaziz I. Makertihartha I. G. B. N. Subagjo L. aniwati M. Al-Betar A.-R. (2019). Fabrication zone of OSDA-free and seed-free mordenite crystals. Powder Technol. 342, 992–997. 10.1016/j.powtec.2018.09.041

[B34] Jamil A. K. Muraza O. Ahmed M. H. Zainalabdeen A. Muramoto K. Nakasaka Y. (2018). Hydrothermally stable acid-modified ZSM-22 zeolite for selective propylene production via steam-assisted catalytic cracking of n-hexane. Micropor. Mesopor. Mater. 260, 30–39. 10.1016/j.micromeso.2017.10.016

[B35] Jamil A. K. Muraza O. Osuga R. Shafei E. N. Choi K.-H. Yamani Z. H. (2016). Hydrothermal stability of one-dimensional pore ZSM-22 zeolite in hot water. J. Phys. Chem. C 120, 22918–22926. 10.1021/acs.jpcc.6b04980

[B36] Jang H.-S. Bae K. Shin M. Kim S. M. Kim C.-U. Suh Y.-W. (2014). Aromatization of glycerol/alcohol mixtures over zeolite H-ZSM-5. Fuel 134, 439–447. 10.1016/j.fuel.2014.05.086

[B37] Khalil U. Muraza O. (2016). Microwave-assisted hydrothermal synthesis of mordenite zeolite: optimization of synthesis parameters. Micropor. Mesopor. Mater. 232, 211–217. 10.1016/j.micromeso.2016.06.016

[B38] Kowalska-Kus J. Held A. Frankowski M. Nowinska K. (2017). Solketal formation from glycerol and acetone over hierarchical zeolites of different structure as catalysts. J. Mol. Catal. A Chem. 426, 205–212. 10.1016/j.molcata.2016.11.018

[B39] Kurniawan T. Muraza O. Hakeem A. S. Al-Amer A. M. (2017a). Mechanochemical route and recrystallization strategy to fabricate mordenite nanoparticles from natural zeolites. Cryst. Growth Des. 17, 3313–3320. 10.1021/acs.cgd.7b00295

[B40] Kurniawan T. Muraza O. Miyake K. Hakeem A. S. Hirota Y. Al-Amer A. M. (2017b). Conversion of dimethyl ether to olefins over nanosized mordenite fabricated by a combined high-energy ball milling with recrystallization. Ind. Eng. Chem. Res. 56, 4258–4266. 10.1021/acs.iecr.6b04834

[B41] Lauriol-Garbay P. Millet J. M. M. Loridant S. Bellière-Baca V. Rey P. (2011). New efficient and long-life catalyst for gas-phase glycerol dehydration to acrolein. J. Catal. 280, 68–76. 10.1016/j.jcat.2011.03.005

[B42] Li L. Korányi T. I. Sels B. F. Pescarmona P. P. (2012). Highly-efficient conversion of glycerol to solketal over heterogeneous lewis acid catalysts. Green Chem. 14, 1611–1619. 10.1039/c2gc16619d

[B43] Mahdi H. I. Irawan E. Nuryoto N. Jayanudin J. Sulistyo H. Sediawan W. B. (2016). Glycerol carbonate production from biodiesel waste over modified natural clinoptilolite. Waste Biomass Valorization 7, 1349–1356. 10.1007/s12649-016-9495-3

[B44] Manjunathan P. Maradur S. P. Halgeri A. B. Shanbhag G. V. (2015). Room temperature synthesis of solketal from acetalization of glycerol with acetone: effect of crystallite size and the role of acidity of beta zeolite. J. Mol. Catal. A Chem. 396, 47–54. 10.1016/j.molcata.2014.09.028

[B45] Marimuthu M. Marimuthu P. S. K. A. K. Palanivelu S. Rajagopalan V. (2018). Tuning the basicity of Cu-based mixed oxide catalysts towards the efficient conversion of glycerol to glycerol carbonate. Mol. Catal. 460, 53–62. 10.1016/j.mcat.2018.09.002

[B46] Monteiro M. R. Kugelmeier C. L. Pinheiro R. S. Batalha M. O. Da Silva César A. (2018). Glycerol from biodiesel production: technological paths for sustainability. Renew. Sustain. Energy Rev. 88, 109–122. 10.1016/j.rser.2018.02.019

[B47] Muraza O. Bakare I. A. Tago T. Konno H. Adedigba A.-L. Al-Amer A. M. (2013). Controlled and rapid growth of MTT zeolite crystals with low-aspect-ratio in a microwave reactor. Chem. Eng. J. 226, 367–376. 10.1016/j.cej.2013.04.072

[B48] Muraza O. Bakare I. A. Tago T. Konno H. Taniguchi T. Al-Amer A. M. (2014). Selective catalytic cracking of n-hexane to propylene over hierarchical MTT zeolite. Fuel 135, 105–111. 10.1016/j.fuel.2014.06.045

[B49] Nanda M. R. Zhang Y. Yuan Z. Qin W. Ghaziaskar H. S. Xu C. (2016). Catalytic conversion of glycerol for sustainable production of solketal as a fuel additive: a review. Renew. Sustain. Energy Rev. 56, 1022–1031. 10.1016/j.rser.2015.12.008

[B50] Nandiwale K. Y. Patil S. E. Bokade V. V. (2014). Glycerol etherification using n-butanol to produce oxygenated additives for biodiesel fuel over H-beta zeolite catalysts. Energy Technol. 2, 446–452. 10.1002/ente.201300169

[B51] Nasser G. Kurniawan T. Miyake K. Galadima A. Hirota Y. Nishiyama N. (2016a). Dimethyl ether to olefins over dealuminated mordenite (MOR) zeolites derived from natural minerals. J. Nat. Gas Sci. Eng. 28, 566–571. 10.1016/j.jngse.2015.12.032

[B52] Nasser G. A. Kurniawan T. Tago T. Bakare I. A. Taniguchi T. Nakasaka Y. (2016b). Cracking of n-hexane over hierarchical MOR zeolites derived from natural minerals. J. Taiwan Inst. Chem. Eng. 61, 20–25. 10.1016/j.jtice.2015.11.025

[B53] Nasser G. A. Muraza O. Nishitoba T. Malaibari Z. Al-Shammari T. K. Yokoi T. (2019). OSDA-free chabazite (CHA) zeolite synthesized in the presence of fluoride for selective methanol-to-olefins. Micropor. Mesopor. Mater. 274, 277–285. 10.1016/j.micromeso.2018.07.020

[B54] Pawar R. R. Gosai K. A. Bhatt A. S. Kumaresan S. Lee S. M. Bajaj H. C. (2015). Clay catalysed rapid valorization of glycerol towards cyclic acetals and ketals. RSC Adv. 5, 83985–83996. 10.1039/C5RA15817F

[B55] Pentrák M. Hronský V. Pálková H. Uhlík P. Komadel P. Madejová J. (2018). Alteration of fine fraction of bentonite from Kopernica (Slovakia) under acid treatment: a combined XRD, FTIR, MAS NMR and AES study. Appl. Clay Sci. 163, 204–213. 10.1016/j.clay.2018.07.028

[B56] Popova M. Lazarova H. Kalvachev Y. Todorova T. Szegedi Á. Shestakova P. (2017). Zr-modified hierarchical mordenite as heterogeneous catalyst for glycerol esterification. Catal. Commun. 100, 10–14. 10.1016/j.catcom.2017.06.009

[B57] Possato L. G. Chaves T. F. Cassinelli W. H. Pulcinelli S. H. Santilli C. V. Martins L. (2017). The multiple benefits of glycerol conversion to acrolein and acrylic acid catalyzed by vanadium oxides supported on micro-mesoporous MFI zeolites. Catal. Today 289, 20–28. 10.1016/j.cattod.2016.08.005

[B58] Presciutti A. Asdrubali F. Baldinelli G. Rotili A. Malavasi M. Di Salvia G. (2018). Energy and exergy analysis of glycerol combustion in an innovative flameless power plant. J. Clean. Prod. 172, 3817–3824. 10.1016/j.jclepro.2017.06.022

[B59] Prinsen P. Luque R. González-Arellano C. (2018). Zeolite catalyzed palmitic acid esterification. Micropor. Mesopor. Mater. 262, 133–139. 10.1016/j.micromeso.2017.11.029

[B60] Quispe C. A. G. Coronado C. J. R. Carvalho J. A. Jr (2013). Glycerol: Production, consumption, prices, characterization and new trends in combustion. Renew. Sustain. Energy Rev. 27, 475–493. 10.1016/j.rser.2013.06.017

[B61] Ren21 T. (2018). “Renewables 2018 Global Status Report,” in: Renewables 2018 Global Status Report (GSR). The REN21 Network.

[B62] Samudrala S. P. Kandasamy S. Bhattacharya S. (2018). Turning biodiesel waste glycerol into 1,3-Propanediol: catalytic performance of sulphuric acid-activated montmorillonite supported platinum catalysts in glycerol hydrogenolysis. Sci. Rep. 8:7484. 10.1038/s41598-018-25787-w 29749394 PMC5945670

[B63] Sandesh S. Halgeri A. B. Shanbhag G. V. (2015). Utilization of renewable resources: condensation of glycerol with acetone at room temperature catalyzed by organic–inorganic hybrid catalyst. J. Mol. Catal. A Chem. 401, 73–80. 10.1016/j.molcata.2015.02.015

[B64] Sanhoob M. A. Muraza O. Shafei E. N. Yokoi T. Choi K.-H. (2017). Steam catalytic cracking of heavy naphtha (C12) to high octane naphtha over B-MFI zeolite. Appl. Catal. B Environ. 210, 432–443. 10.1016/j.apcatb.2017.04.001

[B65] Sanhoob M. A. Muraza O. Taniguchi T. Tago T. Watanabe G. Masuda T. (2016). Steam catalytic cracking of n-hexane over modified MTW zeolites impregnated by extra-framework elements. Energy Fuels 30, 9679–9685. 10.1021/acs.energyfuels.6b00857

[B66] Stocker K. Ellersdorfer M. Lehner M. Raith J. G. (2017). Characterization and utilization of natural zeolites in technical applications. BHM Berg und Hüttenmännische Monatshefte 162, 142–147. 10.1007/s00501-017-0596-5

[B67] Tamiyakul S. Ubolcharoen W. Tungasmita D. N. Jongpatiwut S. (2015). Conversion of glycerol to aromatic hydrocarbons over Zn-promoted HZSM-5 catalysts. Catalysis Today 256, 325–335. 10.1016/j.cattod.2014.12.030

[B68] Tan H. W. Abdul Aziz A. R. Aroua M. K. (2013). Glycerol production and its applications as a raw material: a review. Renew. Sustain. Energy Rev. 27, 118–127. 10.1016/j.rser.2013.06.035

[B69] Wang F. Chu X. Zhu F. Wu F. Li Q. Liu B. (2019). Producing BTX aromatics-enriched oil from biomass derived glycerol using dealuminated HZSM-5 by successive steaming and acid leaching as catalyst: reactivity, acidity and product distribution. Micropor. Mesopor. Mater. 277, 286–294. 10.1016/j.micromeso.2018.11.015

[B70] Wang F. Xiao W. Gao L. Xiao G. (2016). Enhanced performance of glycerol to aromatics over Sn-containing HZSM-5 zeolites. RSC Adv. 6, 42984–42993. 10.1039/C6RA03358J

[B71] Wang F. Zhou M.-X. Yang X.-H. Gao L.-J. Xiao G.-M. (2017). The effect of hierarchical pore architecture on one-step catalytic aromatization of glycerol: Reaction routes and catalytic performances. Mol. Catal. 432, 144–154. 10.1016/j.mcat.2017.01.017

[B72] Xiao Y. Varma A. (2016). Conversion of glycerol to hydrocarbon fuels via bifunctional catalysts. ACS Energy Lett. 1, 963–968. 10.1021/acsenergylett.6b00421

[B73] Yang X. Wang F. Wei R. Li S. Wu Y. Shen P. (2018). Synergy effect between hierarchical structured and Sn-modified H[Sn, Al]ZSM-5 zeolites on the catalysts for glycerol aromatization. Micropor. Mesopor. Mater. 257, 154–161. 10.1016/j.micromeso.2017.08.039

[B74] Yu Q. Cui C. Zhang Q. Chen J. Li Y. Sun J. (2013). Hierarchical ZSM-11 with intergrowth structures: synthesis, characterization and catalytic properties. J. Energy Chem. 22, 761–768. 10.1016/S2095-4956(13)60101-1

[B75] Zakaria Z. Y. Linnekoski J. Amin N. A. S. (2012). Catalyst screening for conversion of glycerol to light olefins. Chem. Eng. J. 207–208, 803–813. 10.1016/j.cej.2012.07.072

[B76] Zhang H. Hu Z. Huang L. Zhang H. Song K. Wang L. (2015). Dehydration of glycerol to acrolein over hierarchical ZSM-5 zeolites: effects of mesoporosity and acidity. ACS Catal. 5, 2548–2558. 10.1021/cs5019953

